# Outlines of Pathology and Treatment of Syphilis and Other Venereal Diseases

**Published:** 1887-03

**Authors:** 


**Outlines of the Pathology and Treatment of Syphilis and other Venereal Diseases.** By H. VON ZEISSL, M. D., late Professor at the Royal University, Vienna. Second edition. Revised by M. Von Zeissl; authorized translation and notes by H. Raphael, M. D., Bellevue Hospital. New York: D. Appleton & Co. 1886.

As a clinical observer and teacher of venereal diseases, Professor Von Zeissl has occupied a first rank in Europe. The appearance in this country of the translations of his work on syphilis was cordially welcomed. The concise and graphic descriptions of the various forms of the disease, the accurate delineations of the pathological lesions, the terse and detailed account of the symptomatology, made it one of the most valuable guides in the study of venereal affections. This second edition has been improved in many ways. It is divided into three sections: I. Gonorrhœa; II. Chancroid; III. Syphilis. To the general practitioner it presents a brief graphic and comprehensible picture of these diseases, and will be found an invaluable aid to the proper diagnosis and treatment of the various venereal affections. The publishers have issued the book in most handsome form.

